# Innovative chemistry research driven by medicine

**DOI:** 10.1016/j.fmre.2023.05.006

**Published:** 2023-05-25

**Authors:** Hang Yin, Chunhai Fan, Catherine Chiulan Wong, Lanqun Mao, Yanyi Huang, Guo-Qiang Chen

**Affiliations:** a School of Pharmaceutical Sciences, Tsinghua University, Beijing 100084, China; b School of Chemistry and Chemical Engineering, New Cornerstone Science Laboratory, Frontiers Science Center for Transformative Molecules and National Center for Translational Medicine, Shanghai Jiao Tong University, Shanghai 200240, China; c Clinical Research Institute, State Key Laboratory of Complex Severe and Rare Diseases, Peking Union Medical College Hospital, Chinese Academy of Medical Science & Peking Union Medical College, Beijing 100730, China; d College of Chemistry, Beijing Normal University, Beijing 100875, China; e College of Chemistry and Molecular Engineering, Beijing National Laboratory for Molecular Sciences, Biomedical Pioneering Innovation Center, Peking-Tsinghua Center for Life Sciences, Peking University, Beijing 100871, China; f Institute of Aging & Tissue Regeneration, State Key Laboratory of Systems Medicine for Cancer and Chinese Academy of Medical Sciences Research Unit (No. 2019RU043), Ren-Ji Hospital, Shanghai Jiao Tong University School of Medicine, Shanghai 200127, China

Chemistry, a fundamental scientific discipline, plays a significant role in various fields, including medical science. Its ability to break down human cells into constituent biomolecules, such as nucleic acids, proteins, carbohydrates and lipids, has been a valuable resource for studying fundamental building blocks at the atomic level. By studying the functions of these biomolecules, chemists can gain insights into the underlying mechanisms of various diseases. The integration of chemistry and medical science has facilitated advancements in diagnostic, therapeutic and translational research, leading to fruitful collaborations between these two fields. As a result, this interdisciplinary approach has yielded cutting-edge research with implications for the improvement of human health.

On the other hand, chemical synthesis of molecules and biomolecules has been a powerful tool in creating desired products that are difficult to obtain through biological production methods. This approach has resulted in discoveries about the complex machinery of large biomolecules implicated in challenging-to-treat diseases such as autoimmune diseases, cancers, genetic disorders, infectious diseases, and neurodegenerative diseases. Moreover, this technique has facilitated the development of novel molecules for use in diagnostics or drug discovery, bridging the gap between basic research and translational applications. Chemists have applied chemical synthesis techniques to target enzymes and receptors involved in various biological pathways implicated in diseases. In addition, they have designed drug delivery systems, such as nanoparticles, liposomes, and micelles, to improve drug efficacy. Analytical instruments have provided precise and accurate detection, quantification, and characterization of biomolecules in biological systems. Furthermore, they have developed biomaterials through chemical synthesis to repair or replace damaged tissues or organs. Chemical biology has enabled scientists to develop probes that aid in the profiling of new drug targets, thereby facilitating the development of innovative therapies for diseases. The emergence of diseases such as COVID-19 has had a profound impact on the world, endangering lives globally. However, chemists have played a crucial role in combating these diseases by driving the development of vaccines and discovering new drugs.

In the last decade, research laboratories have swiftly incorporated cutting-edge advancements, such as artificial intelligence, computational tools, imaging technologies, novel chemical reactions, genomics, proteomics, metabolomics, and imaging mass spectrometry. The utilization of these technologies has furnished scientists with supplementary tools to tackle previously intractable problems, resulting in a more comprehensive understanding of molecules in the medical science context. Consequently, it is now feasible to explore complex systems and identify critical components that are essential for translational research.

This special issue of *Fundamental Research* includes original research articles, reviews and so on, with the aim of providing novel insights and perspectives on the subject matter. This special issue covers topics from various research fields, including strategies for targeting amyloid proteins in the diagnosis of neurodegenerative diseases [1], next-generation technologies for detecting airborne diseases [2], and in vivo multicolor ^19^F magnetic resonance imaging for precision medicine [3]. This special issue also introduces recent advances and applications of DNA hydrogels [4], single-particle characterization of lipid-based nanomedicines [5], imaging liver glycogen in metabolic disorders [6] and ferroptosis induction by metal complexes for anti-cancer therapy [7]. It is anticipated that this issue will stimulate further discussion and spark new ideas in the field. We would like to extend our gratitude to all the authors who have made significant contributions to this special issue. We would also like to acknowledge the invaluable support and assistance provided by the editorial board members and the editorial office.
